# In Vitro and In Vivo Evaluation of the Effectiveness and Safety of Amygdalin as a Cancer Therapy

**DOI:** 10.3390/ph15111306

**Published:** 2022-10-22

**Authors:** Fatma I. Abo El-Ela, Amr Gamal, Hossny Awad Elbanna, Ahmed H. ElBanna, Heba F. Salem, Alaa S. Tulbah

**Affiliations:** 1 Department of Pharmacology, Faculty of Veterinary Medicine, Beni-Suef University, Beni-Suef 62511, Egypt; 2 Department of Pharmaceutics and Industrial Pharmacy, Faculty of Pharmacy, Beni-Suef University, Beni-Suef 62511, Egypt; 3 Department of Pharmacology, Faculty of Veterinary Medicine, Cairo University, Cairo 11511, Egypt; 4 Michael Sayegh Faculty of Pharmacy, Aqaba University of Technology, Aqaba 77110, Jordan; 5 Department of Pharmaceutics, College of Pharmacy, Umm Al Qura University, Makkah 24451, Saudi Arabia

**Keywords:** cancer, herbal medicine, amygdalin, niosomes, DMBA

## Abstract

Cancer is one of the most important causes of death worldwide. Several studies have shown the efficacy of apricot kernel seed as a cancer therapy due to the presence of amygdalin. These studies have demonstrated amygdalin’s cytotoxicity, antioxidant activity, and apoptosis in vitro using human cancer cell lines. However, no studies have demonstrated their cancer activity in vivo. The aim of this study is to develop an amygdalin-loaded niosomes (ALN) gel formulation as a drug delivery system in order to investigate the selectivity, efficacy, and toxicity of amygdalin as a cancer therapy in vivo using the 7,12-dimethylbenz (a) anthracene (DMBA) carcinoma rat model. Based on pre-formulation studies, the ALN formulation composed of Tween 60: cholesterol: dihexadecyl phosphate in a molar ratio of 1:2:0.1 was chosen as an optimum formulation because it has a percent of EE of 66.52% with a particle size of 269.3 nm and a reflux of 3.54 µg.cm^−2^.h^−1^. The ALN gel formulation was integrated into carbopol gel to be evaluated in vivo. Compared to DMBA control, treatment with ALN gel showed a reduction in the carcinoma volume and in the hyperplasia of the epidermis with no signs of edema. In conclusion, the ALN gel formulation could be an efficient cancer therapy.

## 1. Introduction

“Cancer” is a group of diseases characterized by the uncontrolled growth and metastasis of malignant cells throughout the body [1,2]. Throughout the 20th and 21st centuries, cancer has been one of the most important causes of death [3,4,5]. Numerous cancer treatment options, such as radiation, chemotherapy, and surgery, have been established [6,7]. However, herbal medicine is seen as a possible future cancer therapy due to its low side effects, high pharmacological efficacy, and ability to be obtained at low prices [8,9]. Natural products are a promising resource for chemo-preventive and chemo-therapeutic drug development [10,11,12,13,14,15,16,17,18]. Approximately 80% of all medications approved by the FDA in the last three decades have been derived from natural sources [19,20]. Thus, it is crucial to develop chemo-preventive and chemo-therapeutic drugs derived from nature. Apricot kernel seed has been employed as a model herbal medicine. Several studies have shown the efficacy of using apricot kernel in cancer therapy [1,8,21,22,23,24,25,26]. These studies suggest that the presence of amygdalin in apricot kernels is responsible for their anti-proliferative properties. Amygdalin induces apoptosis, which inhibits cancer cell proliferation and survival [1,8,21]. Amygdalin has cyanogenic glycosides that are broken down by the beta-glucosidase enzyme into hydrogen cyanide and benzaldehyde, which synergistically destroy and kill cancer cells [23,25]. It also has antioxidant properties that reduce oxidative stress in cancer cells [26]. Numerous studies have demonstrated amygdalin’s cytotoxicity and apoptosis in human cancer cell lines [24,27,28,29,30]. However, to the best of our knowledge, no studies have been conducted to demonstrate the efficacy and toxicity of amygdalin in vivo using the 7, 12-dimethylbenz(a) anthracene (DMBA) carcinoma rat model.

Despite the fact that amygdalin has been shown to be effective as a cancer therapy, it can cause cyanide poisoning if taken orally in tablet form [31,32]. Transdermal drug delivery is a promising route for cancer treatment compared with the oral route due to its low side effects and improved efficacy and selectivity [33,34]. No first-pass metabolism or plasma drug level fluctuation is associated with transdermal drug delivery [35,36]. However, the presence of the stratum corneum prevents drugs from being delivered deeply into and across the skin. The inclusion of permeation enhancers can enhance skin permeability and facilitate drug absorption [37,38]. Nano-particles, such as liposomes and niosomes, are created from penetration enhancers that have diffusion properties and could be used to deliver drugs in a targeted and controlled manner [37,38]. Niosomes are better than liposomes because they do not have phospholipids, which can be oxidized and hydrolyzed [37,38]. Noisomes are targeted drug delivery systems that use non-ionic surfactants and cholesterol to increase transdermal transport and efficacy of water-soluble drugs, such as amygdalin [37,38,39,40]. Non-ionic surfactants diminish stratum corneum’s barrier characteristics by altering its partitioning potential and emulsifying the sebum’s composition [37,38]. Noisomes improve the bioavailability and efficacy of drugs in neoplastic cells [37,41]. As a solubilizing matrix, niosomes have been shown to increase drug stability and reduce drug toxicity [37,38,39]. The aim of this study is to develop an amygdalin-loaded niosome (ALN) gel formulation as an efficient drug delivery system for amygdalin in an attempt to study the efficacy and toxicity of amygdalin as a cancer therapy in vivo using the DMBA carcinoma rat model. The design and evaluation of different ALN formulations were set up so that the optimum formulation could be chosen based on criteria, such as enhancing delivery and permeation. Then, the optimum formulation was put into a carbopol gel to investigate the efficacy and toxicity of the ALN gel formulation compared to oral tamoxifen in vivo using the DMBA carcinoma rat model. 

## 2. Results and Discussion

### 2.1. Preparation and In Vitro Characterization of Optimum ALN Formulation

#### 2.1.1. Optimization of ALN Formulations

Different ALN formulations were successfully prepared. The standard calibration curve as described by Sohail et al. was obtained and found to be reliable to quantify amygdalin with a coefficient of determination (R^2^) of 0.999, indicating linearity [29]. Pre-formulation studies were carried out to identify the ability of independent variables to form amygdalin-loaded niosomes [38,41,42,43]. HLB expresses proportionally the strength of polarity of surfactants and allows for the selection of appropriate surfactants to produce physically stable niosomes [39,40,44]. Span 60 and Tween 60 were used as non-ionic surfactants because their lengthy alkyl chains allowed for the production of niosomes with a high percent of EE and rigid vesicular membranes [37,40,43,45]. The percent of EE and particle size of different ALN formulations were determined and found to range from 5.97 ± 1.05% to 66.52 ± 0.57% and from 175.5 ± 17.12 nm to 393.33 ± 8.60 nm, respectively. As shown in Table 1, a rise in HLB values was associated with a statistically significant (*p* < 0.05) increase in the percent of EE, particle size, and PDI. The formulations composed of Tween 60 gave better encapsulation of amygdalin and the highest particle size and PDI due to the hydrophilicity of amygdalin, high HLB, and high surface free energy of Tween 60, allowing for the formation of stiff large vesicles [37,39,40,43]. As the value of HLB was reduced, the percent of EE, PDI, and size of the vesicles decreased due to the high hydrophobicity of Span 60 [43]. These findings were consistent with those of Waddad et al. and Nowroozi et al. [39,43]. Cholesterol is a stiff molecule that increases the bilayer’s rigidity and, consequently, its physical stability [37,39,43,44]. The results of pre-formulation studies revealed that a rise in cholesterol content of the formulations composed of Tween 60 was associated with a statistically significant (*p* < 0.05) increase in the percent of EE and a decrease in particle size and PDI. These results were obtained because cholesterol decreases the surface free energy and enhances the bilayer hydrophobicity and rigidity, leading to fewer leaky and stable vesicles [37,39,43,44]. These findings were consistent with those of Chaw et al. and Waddad et al. [41,43]. DDP is a charge inducer used in the preparation of niosomes to give them a high negative zeta-potential value and, thus, repulsive forces with the skin surface, which is beneficial for stability and transdermal drug delivery [40,43]. All ALN formulations containing DDP exhibited a higher percent of EE and smaller particle size and PDI than those that did not have DDP at the molar ratio investigated. However, by increasing the amount of DDP beyond the limit, a significant (*p* < 0.05) decrease in the percent of EE and a significant (*p* < 0.05) increase in the particle size and PDI were observed because DDP increases the surface free energy and the bilayer hydrophilicity [40,41,43,46,47]. These findings were consistent with those of Waddad et al. [43]. According to these outcomes, the formulation composed of Tween 60: cholesterol: DDP in a molar ratio of 1:2:0.1 was considered the formulation of choice because it had a high percent of EE of 66.52 ± 0.57% with a small particle size of 269.3 ± 9.58 nm and a low PDI of 0.331 ± 0.01.

#### 2.1.2. Differential Scanning Calorimetry (DSC)

**Figure 1:**
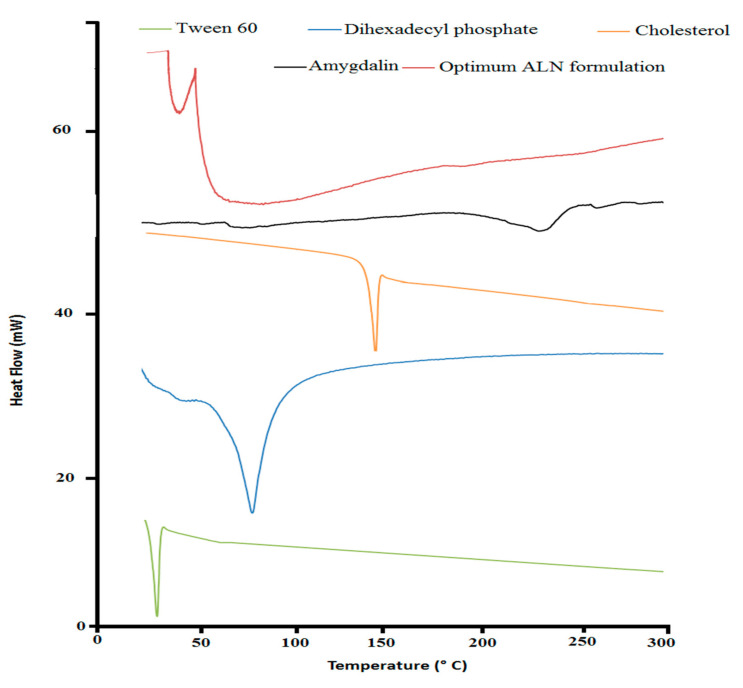
DSC of optimum ALN formulation and its individual constituent.

Thermograms of pure amygdalin, a lyophilized optimum ALN formulation, Tween 60, cholesterol, and DDP, are shown in Figure 1. The DSC curves of amygdalin_,_ Tween 60, DDP, and cholesterol revealed sharp endothermic peaks at 227 °C, 25 °C, 79 °C, and 149 °C, respectively, corresponding to their melting points. The DSC thermogram of the optimum ALN formulation manifested the shifting of the endotherms of Tween 60 and the disappearance of the characteristic peaks of amygdalin, DDP, and cholesterol. The DSC was used to highlight the impact of the niosomal formulation on their ingredients and amygdalin by altering their thermodynamic characteristics [48,49]. Shifting the thermal peak of Tween 60 indicated an alteration of the transition peak of Tween 60 [41,48,49]. The high percentage of EE of ALN and complete solubility of amygdalin could explain the absence of the amygdalin thermal peak.

#### 2.1.3. Transmission Electron Microscopy (TEM) 

**Figure 2:**
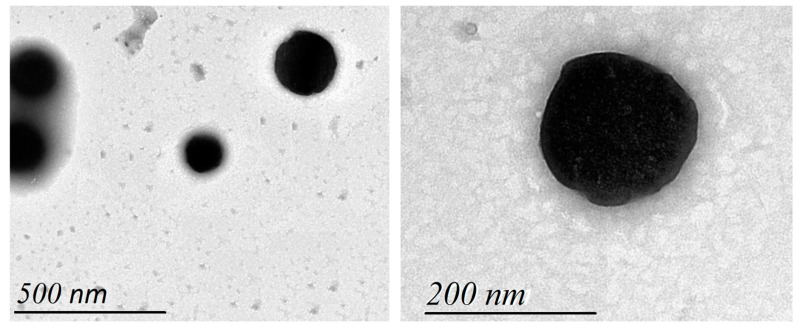
Transmission electron microscopy of optimum ALN formulation.

The morphology of the optimum ALN formulation was investigated in Figure 2. The vesicles showed spherical vesicular structures existing in a dispersed pattern.

#### 2.1.4. Zeta Potential

**Figure 3:**
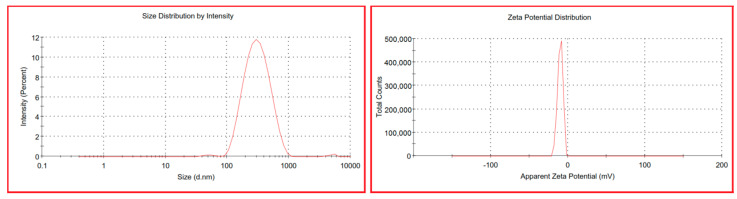
Particle size and zeta potential of optimum ALN formulation.

The PDI and zeta potential of the optimum ALN formulation were investigated in Figure 3. The optimum ALN formulation had a low PDI value, indicating a consistent size distribution, low interfacial tension, a homogeneous noisome, and fewer tendencies for aggregation [39,40,43]. It is crucial to measure the electrostatic charge of the optimum ALN formulation to assess its stability and potential for transdermal drug administration [40,41]. The zeta potential of the optimum ALN formulation was found to be −4.88 ± 0.65. The value of the zeta potential indicated a negative surface charge, which is considered advantageous for transdermal drug delivery and for electrostatic stabilization due to the electrostatic repulsions between vesicles [40,41,50,51].

#### 2.1.5. In Vitro Amygdalin Release Kinetics Study 

**Figure 4:**
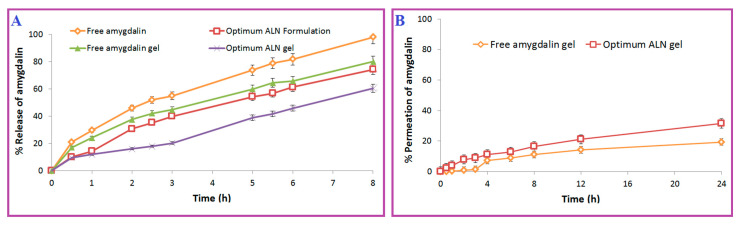
Release (**A**) and permeation (**B**) profiles of optimum ALN gel formulation.

Based on the pre-formulation study, 20 mL of PB (pH 7.4) was used as a release medium because it was greater than the saturation solubility of amygdalin and could be used in the sink condition of the release and permeation studies. These findings were consistent with those of Sohail et al. [29]. It was obtained from the release profile (Figure 4A) that amygdalin was released from the optimum ALN formulation at a lower rate (*p* < 0.05) compared with free amygdalin due to the high cholesterol content and the presence of DDP [38,43]. These findings were consistent with those of Waddad et al. [43]. In Table 2, the kinetics of the release of amygdalin from the optimum ALN formulation were compared with free amygdalin. The data of the optimum ALN formulation best fitted the Weibull model, while the data of free amygdalin best fitted the Korsmeyer–Peppas model because they had minimum AIC and maximum R^2^ and MSC. Non-fickian diffusion was shown to be the mechanism of release for both the optimum ALN formulation (n = 0.704) and the amygdalin solution (n = 0.565). Dissolution profiles of both the optimum ALN formulation and the amygdalin solution were also observed to differ significantly (*p* < 0.05), with *f*_2_ being 42.46.

### 2.2. Preparation and In Vitro Characterization of Optimum ALN Gel Formulation

#### 2.2.1. Viscosity Coefficient Measurement of Optimum ALN Gel Formulation

Successfully, optimum ALN formulation and free amygdalin gel formulations were prepared. Carbopol^®^ is a crosslinked anionic synthetic polymer with high viscosity and bio-adhesive properties [48,50,52]. Triethanolamine was used as a neutralizing agent [52]. The optimum ALN gel formulation had a viscosity that was statistically (*p* < 0.05) higher than that of the free amygdalin gel. Viscosity coefficient measurement (Table 3) showed that the viscous characters of the optimum ALN formulation were increased due to the crosslinking of carbopol polymer and niosome alignment as a function of shear stress [48]. 

#### 2.2.2. Release and Permeation Studies of Optimum ALN Gel Formulation

It was obtained from the permeation profile (Figure 4B) that amygdalin was permeated from the optimum ALN gel at a higher rate (*p* < 0.05) compared with free amygdalin gel_._ Table 3 displays the release and flux of the optimum ALN gel formulation compared to that of the free amygdalin gel. The crosslinking and high viscosity of carbopol polymer explain the slow release of the prepared gels [48,50]. The presence of a non-ionic surfactant (tween 60) in the structure of niosomes was correlated with improved permeation due to its ability to be adsorbed at interfaces and its ability to modify the lipid membranes by disrupting the stratum corneum barrier [48,53]. These findings were consistent with those of Arafa et al. [48]. The kinetics of release of amygdalin from the optimum ALN gel best fitted the zero model, while data of free amygdalin gel best fitted the Korsmeyer–Peppas model because they had minimum AIC and maximum R^2^ and MSC. Non-fickian diffusion was shown to be the mechanism of release for both the optimum ALN gel (n = 0.940) and the amygdalin gel (n = 0.553).

### 2.3. In Vivo Characterization of Optimum ALN Gel Formulation

#### 2.3.1. Treatment Efficiency of Optimum ALN Gel Formulation

**Figure 5:**
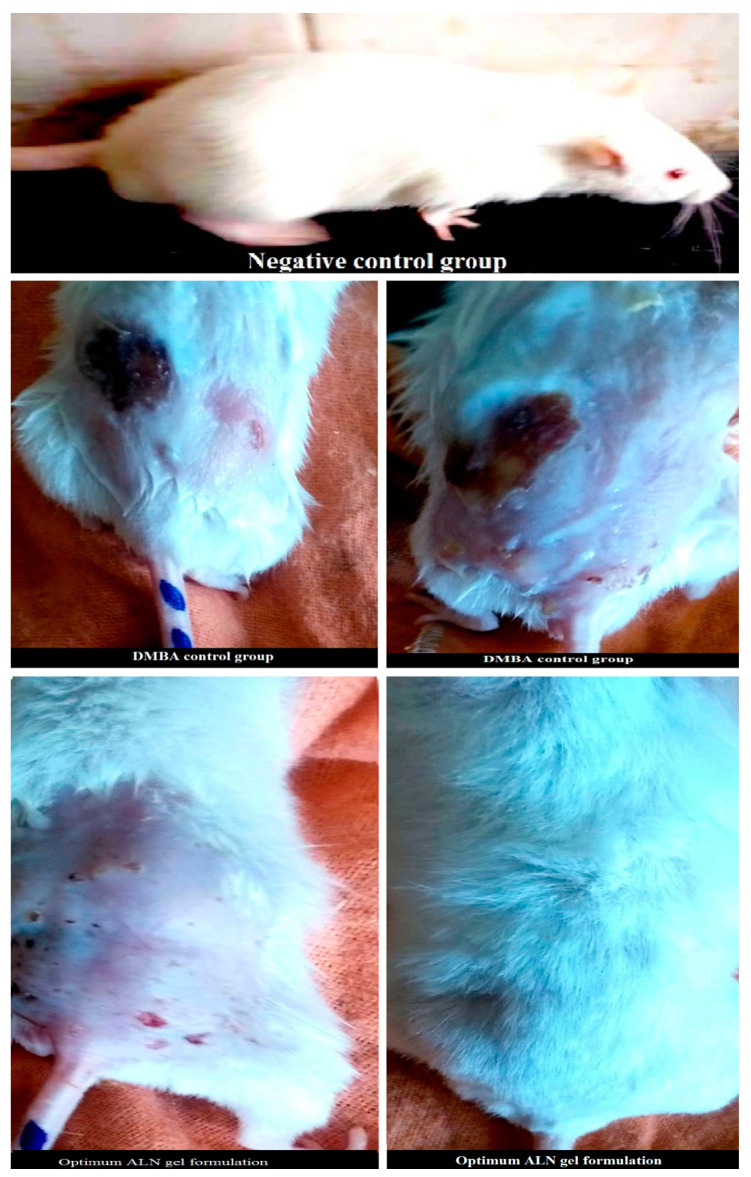
Representative images of carcinoma in optimum ALN gel formulation treated rats compared to DMBA control.

**Figure 6:**
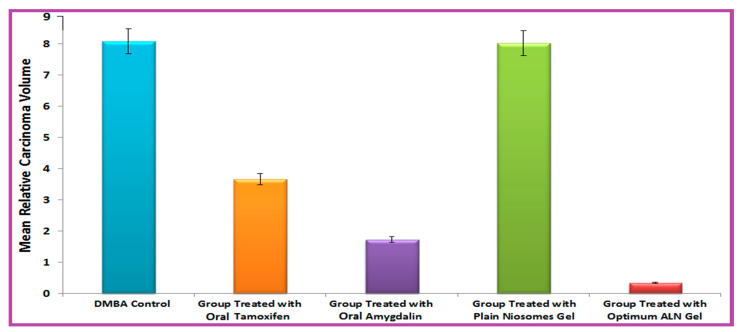
Treatment efficiency of optimum ALN gel formulation.

It was obtained from Figure 5 that the optimum ALN gel formulation reduced mean relative carcinoma volume (MCV) compared with the DMBA control. The efficiency of treatment (Figure 6) was in the following arrangement: optimum ALN gel > oral amygdalin > oral tamoxifen > plain niosomes gel. The optimum ALN gel formulation reduced mean relative carcinoma volume (MCV) at a higher rate (*p* < 0.05) compared with free amygdalin solution and free tamoxifene suspension. The group treated with plain niosomes gel showed MCV nearly similar to that of the DMBA control group. After completing the experiment, the carcinoma growth inhibition (%CGI) of oral tamoxifen, oral amygdalin, and optimum ALN gel was calculated and found to be 54.57, 78.56, and 91.26%, respectively. The group treated with the optimum ALN gel formulation showed the lowest MCV and the highest percent of CGI because niosomes enhanced the permeation of amygdalin and specifically targeted and accumulated it in a solid tumor, creating a drug depot at the site of application and allowing for a controlled release for an extended period of time.

#### 2.3.2. Anti-Tumor Activity of Optimum ALN Gel Formulation

**Figure 7:**
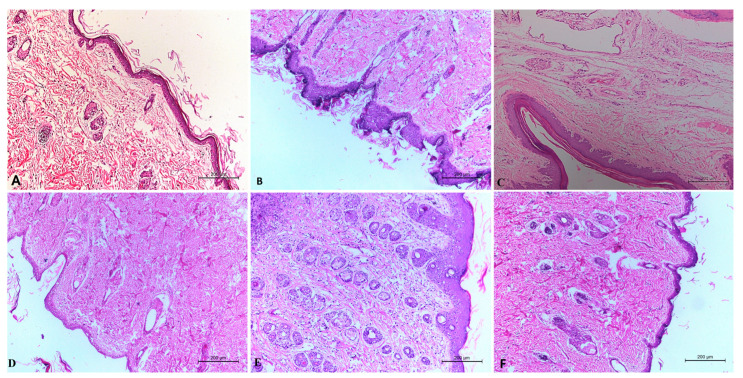
Histolopathological evaluation of negative control group (**A**), DMBA control (**B**), free tamoxifen suspension (**C**), free amygdalin solution (**D**), plain niosomes gel (**E**), and optimum ALN gel (**F**).

Histological examination of the DMBA control group (Figure 7B) revealed the presence of neoplastic cells, dermal granulation, sub-cutaneous edema, hyperkeratosis, and inflammatory cell infiltrations. Histological examination of the plain niosomes gel treated group (Figure 7E) revealed the presence of all toxicity signs in the skin layers. Histological examination of the oral tamoxifen suspension treated group (Figure 7C) revealed the presence of hyperkeratosis and acanthosis in the surface epithelium of the epidermis with signs of a diffuse inflammatory response and edema in the dermis and sub-cutaneous tissue. Histological examination of the oral amygdalin solution treated group (Figure 7D) observed a reduction in the hyperplasia and acanthosis in the surface epithelium of the epidermis with low signs of a diffuse inflammatory response and edema in the dermis and subcutaneous tissue. Histological examination of the optimum ALN gel treated group (Figure 7F) showed clearly healed skin with normal covering epithelium and marked improvement in all signs of the epidermis and dermis that were better than those of the oral amygdalin solution. These results confirmed the effectiveness of amygdalin loaded niosomes gel as a cancer therapy in vivo.

#### 2.3.3. Toxicity of Optimum ALN Gel Formulation

**Figure 8:**
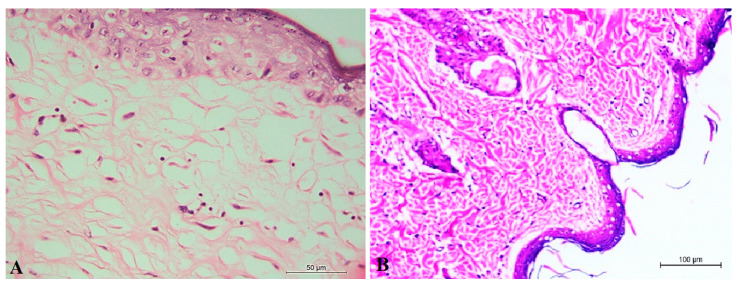
Histolopathological evaluation of negative control group (**A**) and optimum ALN gel (**B**).

Histological examination of the control negative (non-infected or treated rats) group (Figure 8A) showed that the dermal and epidermal layers of the skin were completely normal, and there were no signs of a diffuse inflammatory response or swelling in the dermis and subcutaneous tissue. Histological examination of the optimum ALN gel-treated group (Figure 8B) showed clearly healed skin with normal covering epithelium. A decrease in the surface epithelium and appendages was markedly observed with the appearance of the normal skin structure, which indicated the safety of optimum ALN gel treatment.

## 3. Materials and Methods

### 3.1. Materials

Amygdalin was attained from Nature’s Only Choice Company (Tbilisi, GA, USA). Sigma Aldrich (Agitech Company, Cairo, Egypt) provided Tween 60, Span 60, cholesterol, 7, 12-dimethylbenz[a] anthracene (DMBA), triethanolamine, and dihexadecyl phosphate. Carbopol 934, methanol, acetone, and chloroform were attained from Corner-Lab Company (Cairo, Egypt).

### 3.2. Preparation and In Vitro Characterization of Optimum Amygdalin-Loaded Niosomes (ALN) Formulation

#### 3.2.1. Preparation of ALN Formulations

Pre-formulation studies were carried out to select the optimum ALN formulation for in vitro and in vivo characterization. Different ALN formulations (Table 1) were prepared to study the effects of hydrophilic-lipophilic balance (HLB) values (4.7–14.9), non-ionic surfactant: cholesterol molar ratios (0.5–2), and dihexadecyl phosphate (DDP): non-ionic surfactant molar ratios (0–0.4) as independent variables [38,41,42,43]. HLB expresses proportionally the strength of polarity of surfactants and allows for the selection of appropriate surfactants to produce physically stable niosomes [39,40,44]. Span 60 (HLB = 4.7) and Tween 60 (HLB = 1 4.9) were used as non-ionic surfactants [37,40,43,45]. The particle size and entrapment efficiency (percent of EE) were used as dependent variables. Using the criteria of maximum percent of EE and minimal particle size, optimization was achieved.

Using the thin film hydration method, various ALN formulations were prepared [38]. An organic solution (10 mL) of chloroform and methanol (3:1) was used to dissolve the calculated amounts of non-ionic surfactant, cholesterol, and DDP. This solution was then poured into a round-bottom flask and evaporated under vacuum using a Stuart rotary evaporator (RE300, UK) at 100 rpm and 40 °C. After evaporation of the organic solution, a thin film of niosomes was formed inside the flask. Amygdalin (10 mg) was dissolved in phosphate buffer (PB, 10 mL) and added to the resultant film at 60 °C for 2 h to obtain the ALN formulation. The prepared formulation was sonicated for 30 min with an ultrasonicator (Sonix, IL, USA) and kept at 4 °C.

#### 3.2.2. Determination of Entrapment Efficiency

Using a UV/Vis spectrophotometer at 255 nm, a standard calibration curve was created to measure the amount of amygdalin in an unknown sample. The content of amygdalin entrapped in each ALN formulation was computed by measuring the percent of EE (Equation (1)) [54]. A centrifuge (SIGMA, Steinheim, Germany) was used to isolate ALN pellets from the supernatant at 15,000 rpm for 1 h. The amount of amygdalin in the supernatant was measured using a UV/Vis spectrophotometer at 255 nm in three replicates.

(1) $$\%EE=\frac{\left(\mathrm{Initial}\;\mathrm{amygdalin}\;\mathrm{amount}-\;\mathrm{The}\;\mathrm{amount}\;\mathrm{of}\;\mathrm{amygdalin}\;\mathrm{in}\;\mathrm{the}\;\mathrm{supernatant}\right)}{\mathrm{Initial}\;\mathrm{amygdalin}\;\mathrm{amount}}\times 100$$

#### 3.2.3. Particle Size and Poly Dispersity Index Determination

The particle size and polydispersity index (PDI) are important noisome properties that affect the particle’s dispersion, homogeneity, distribution and subsequent ability to be targeted [39]. Each ALN formulation (1 mL) was diluted with distilled water (9 mL) and measured three times using dynamic light scattering (DLS, Malvern, Germany) to estimate its particle size and PDI.

#### 3.2.4. Differential Scanning Calorimetry (DSC)

Thermal behavior and compatibility of the optimum ALN formulation with its individual constituents were observed by DSC (60F3, Maia, Germany) [55]. Samples (3–5 mg) of amygdalin, optimum ALN formulation, Tween 60, cholesterol, and DDP were put into DSC aluminium pans (50 µL) with a 0.1 mm thickness. DSC thermograms were performed at a heating rate of 5 °C/min, a 25 mL/min flow rate of nitrogen gas, and over a temperature range of 20–300 °C.

#### 3.2.5. Transmission Electron Microscopy (TEM)

The morphology of the optimum ALN formulation and its surface characteristics were observed by TEM (Carl Zeiss, Germany) [50]. A sample (20 µL) of the optimum ALN formulation was applied to a carbon-coated copper grid and stained with phosphotungstic dye. The sample was left to dry and was figured at different magnifications using TEM (70 kV voltages).

#### 3.2.6. Zeta Potential Determination

The zeta potential was determined to measure the electrostatic charge and stability of the optimum ALN formulation [40,41]. The optimum ALN formulation (1 mL) was diluted with distilled water (9 mL) and measured three times using DLS to estimate its zeta potential [50].

#### 3.2.7. In Vitro Amygdalin Release Kinetics Study

The amount of amygdalin released from the optimum ALN formulation was computed by measuring the percent of release compared to free amygdalin (Equation (2)) [29]. Three samples (equivalent to 2 mg of amygdalin) of the optimum ALN formulation were placed in a glass tube with dialysis bags (diffusion membrane with a molecular weight cut-off of 12,000–14,000 Da) covering their lower end. The glass tube was hung up in the Hanson dissolution apparatus and immersed in PB (20 mL, pH 7.4) as a release medium. The experiment was carried out at 100 rpm and 37 ± 0.5 °C to maintain sink conditions. At different times, 2 mL samples were taken and analyzed with a UV/Vis spectrophotometer at 255 nm. The samples were then replaced with the same volume of PB.

(2) $$\%\;Release=\frac{\mathrm{Concentration}\;\mathrm{of}\;\mathrm{amygdalin}\;\mathrm{at}\;\mathrm{each}\;\mathrm{time}\;\mathrm{interval}}{\mathrm{Initial}\;\mathrm{concentration}\;\mathrm{of}\;\mathrm{amygdalin}}\times 100$$

The kinetics of amygdalin’s release from the optimum ALN formulation compared to free amygdalin were determined using DDSolver program software [56]. The release data of the optimum ALN formulation and free amygdalin were analyzed to select the best-fitted model attaining the lowest Akaike information criterion (AIC), the highest model selection criterion (MSC), and the coefficient of determination (R^2^). According to the Korsmeyer–Peppas equation, the mechanism of amygdalin’s release from the optimum ALN formulation compared to free amygdalin was assessed [43]. If the value of *n* is less than 0.5, the release mechanism is fickian; if *n* is between 0.5 and 1, the release mechanism is non-fickian. According to a similarity factor “f_2_”, the significance of the difference between the optimum ALN formulation and free amygdalin was assessed.

### 3.3. Preparation and In Vitro Characterization of Optimum ALN Gel Formulation

#### 3.3.1. Preparation of Optimum ALN Gel Formulation

The optimum ALN gel formulation was prepared using carbopol 934 gel base. The gel was attained by slowly adding 2 gm of carbopol 934–100 mL of water with continuous swirling. The resultant gel was neutralized by triethanolamine [48]. Free amygdalin gel was prepared by slowly stirring free amygdalin into carbopol gel. The optimum ALN gel formulation was prepared by slowly stirring the optimum ALN formulation into carbopol gel.

#### 3.3.2. Viscosity Coefficient Measurement

The viscosity coefficient of the optimum ALN gel formulation compared to free amygdalin gel was measured using a Brookfield viscometer (DV-III ULTRA, USA) [48]. Samples (1 gram) of free amygdalin and ALN gel formulations were placed in a viscometer plate and examined at 37 °C in three replicates. Each run of the Brookfield viscometer involved changing the speed from 5 to 50 rpm, and then the speed was reversed. In order to calculate the viscosity coefficient, the following formula was used:

Log (shear stress) = N log (shear rate) − log (viscosity coefficient)(3)

#### 3.3.3. Ex-Vivo Permeation Study 

The amount of amygdalin permeated from the optimum ALN gel formulation was compared to free amygdalin gel by measuring the percent of permeation (Equation (4)) [48]. Three samples (equivalent to 2 mg of amygdalin) of the optimum ALN gel formulation were placed in a glass tube with excised skin of rats (diffusion membrane with surface area of 5 cm^2^) covering their lower end. The glass tube was hung up in the Hanson dissolution apparatus and immersed in PB (20 mL, pH 7.4) as a receptor medium. The experiment was carried out at 100 rpm and 37 ± 0.5 °C to maintain sink conditions. At different times, 2 mL samples were taken and analyzed with a UV/Vis spectrophotometer at 255 nm. The samples were then replaced with the same volume of PB. The steady-state flux was determined in triplicate as follows [48]:

(4) $$\%\;Permeation=\frac{\mathrm{Concentration}\;\mathrm{of}\;\mathrm{amygdalin}\;\mathrm{at}\;\mathrm{each}\;\mathrm{time}\;\mathrm{interval}}{\mathrm{Initial}\;\mathrm{concentration}\;\mathrm{of}\;\mathrm{amygdalin}}\times 100$$

Steady-state flux = the permeation rate/the skin area(5)

### 3.4. In Vivo Characterization of Optimum ALN Gel Formulation

#### 3.4.1. Tumor Induction

In different cages, thirty adult male Swiss albino rats (200–300 g) were maintained at standard conditions of temperature (22 ± 2 ºC), humidity (50 ± 5%), food, and water. After 7 days of adaptation, the hair on each rat’s back was removed (3 × 3 cm^2^ surface area). Two days later, DMBA (1 mg in 200 μL acetone) was administered to each rat subcutaneously to induce the carcinoma [57]. DMBA is a highly effective carcinogen, capable of causing mammary carcinoma in rats [58,59,60]. In accordance with Beni-Suef University’s animal ethics committee, this method was approved (BSU-IACUC 022-283).

#### 3.4.2. Study Plan

Thirty rats (6 rats/each group) were distributed at random as follows:

A: DMBA control (no treatments were given)

B: Aqueous dispersion of tamoxifen (10 mg/kg body weight [61]) was administered orally to rats.

C: An aqueous solution of amygdalin (10 mg/kg body weight) was administered orally to rats.

D: Plain niosomal gel was topically applied to rats.

E: The optimum ALN gel formulation (10 mg/kg body weight) was topically applied to rats.

#### 3.4.3. Treatment Efficiency of Optimum ALN Gel Formulation

Measuring the mean relative carcinoma volume (MCV) and percentage carcinoma growth inhibition (%CGI) was the standard method to estimate the effectiveness of the optimum ALN gel formulation as a cancer therapy [54]. The width and length of the carcinoma mass of each rat were measured twice weekly with a digital caliber till the end of the experiment. MCV and %CGI were measured as follows:

(6) Carcinoma volume =[(Width of carcinoma mass) ^2 x length of carcinoma mass]2

(7) $$\mathrm{MCV}\;=\frac{\mathrm{Carcinoma}\;volume\;at\;the\;end\;of\;the\;experiment}{\mathrm{Carcinoma}\;volume\;at\;first\;day\;of\;treatment}$$

(8) $$\%\mathrm{CGI}\;=100\;–\left(100\;\times \;\frac{\;\mathrm{Treated}\;\mathrm{group}´s\;\mathrm{MCV}}{\mathrm{DMBA}\;\mathrm{control}´\;\mathrm{MCV}\;}\;\right)$$

#### 3.4.4. Histopathological Examination of Optimum ALN Gel Formulation 

The effectiveness and toxicity of the optimum ALN gel formulation as a cancer therapy were confirmed using histopathological examination [62]. At the end of the study, each rat (6 rats/each group) was sacrificed by cervical dislocation after IP injection of a mixture (0.1 mg/100 gm) of ketamine (90 mg/kg) and xylazine (5 mg/kg) at a ratio of 1:1 for anesthesia. A carcinoma from each rat was taken and preserved in buffered formalin. Sections (4–6 µm) of carcinoma were cut and mounted on clear and dry glass slides. The obtained slides were stained with hematoxylin and eosin (H and E) for histopathological examination by LEICA (DFC290 HD system digital camera, Heerbrugg, Switzerland) connected to the light microscope [62]. All signs observed in the layers of skin (epidermis, dermis, and subcutaneous tissue) were recorded as parameters of treatment efficiency and toxicity. 

### 3.5. Statistical Analysis

In order to determine statistical significance, the student t-test or ANOVA was used via SPSS with *p* < 0.05.

## 4. Conclusions

Pre-formulation studies were carried out to identify the ability of independent variables to form the novel amygdalin-loaded niosomes (ALN) formulation. The results of pre-formulation studies revealed that a formulation composed of Tween 60: cholesterol: dihexadecyl phosphate in a molar ratio of 1:2:0.1 was considered the formulation of choice because it had the highest %EE with a consistent size distribution. The optimum ALN formulation was integrated into carbapol gel to evaluate the efficacy and toxicity of the optimum ALN gel as a cancer therapy in vivo using the DMBA carcinoma rat model. The optimum ALN gel enhanced the permeation of amygdalin into deep skin layers and showed significant anti-tumor activity compared with oral tamoxifen. In conclusion, the optimum ALN gel formulation is an efficient drug delivery system for amygdalin and an efficient cancer therapy.

## Figures and Tables

**Table 1:** In vitro characterization of ALN gel formulations.

Formulation Code	X_1_	X_2_	X_3_	Y_1_ (%) (Mean ± SD)	Y_2_ (nm) (Mean ± SD)	Y_3_ (Mean ± SD)
F1	14.9	2:1	1:0.1	22.32 ± 0.84	349.80 ± 4.51	0.710 ± 0.01
F2	14.9	1:1	1:0.1	28.82 ± 4.30	330.23 ± 10.05	0.610 ± 0.02
F3	14.9	1:2	0	17.53 ± 1.10	395.33 ± 8.60	0.730 ± 0.02
F4	4.7	1:2	1:0.1	5.97 ± 1.05	175.50 ± 17.12	0.253 ± 0.03
F5	9.8	1:2	1:0.1	46.32 ± 0.84	192.43 ± 2.52	0.303 ± 0.02
F6	14.9	1:2	1:0.1	66.52 ± 0.57	269.30 ± 9.58	0.331 ± 0.01
F7	14.9	1:2	1:0.2	48.76 ± 0.45	343.07 ± 16.07	0.523 ± 0.01

X_1_: HLB; X_2_: non-ionic surfactant: cholesterol molar ratios; X_3_: dihexadecyl phosphate: non-ionic surfactant molar ratios. Y_1_: % EE; Y_2_: vesicle size; Y_3_: Poly Dispersity Index. SD: standard deviation.

**Table 2:** Release Kinetics of optimum ALN formulation.

Models		Formulation Code			
		Free Amygdalin Solution	Optimum ALN	Free Amygdalin Gel	Optimum ALN Gel
**Zero-order**	**R^2^**	0.8306	0.8931	0.8301	0.9813
	**AIC**	74.16	68.9844	70.5037	43.3294
	**MSC**	1.1574	1.7850	1.1545	3.5498
**First-order**	**R^2^**	0.9810	0.9778	0.9719	0.9666
	**AIC**	52.6823	53.2812	52.5032	49.1558
	**MSC**	3.3502	3.3553	2.9546	2.9672
**Higuchi**	**R^2^**	0.9944	0.9099	0.9939	0.8702
	**AIC**	40.2658	67.2772	36.825	62.7321
	**MSC**	4.5919	1.9557	4.5224	1.6095
**Korsmeyer–Peppas**	**R^2^**	0.999	0.9521	0.9985	0.9830
	**AIC**	24.6978	62.9484	24.4751	44.3785
	**MSC**	6.1487	2.3886	5.7574	3.4449
**Weibull**	**R^2^**	0.9843	0.997	0.9920	0.9873
	**AIC**	54.7420	36.8968	43.4813	43.5044
	**MSC**	3.1442	4.9938	3.8568	3.5323
**Hixson–Crowell**	**R^2^**	0.9709	0.9720	0.9477	0.9750
	**AIC**	57.0082	55.5794	58.7300	46.2607
	**MSC**	2.9176	3.1255	2.3319	3.2567

**Table 3:** In vitro characterization of optimum ALN gel formulation.

Formulation Code	Viscosity Coefficient (cP)	Release (%)	Flux (µg.cm^−2^.h^−1^)
**Free amygdalin solution**		98.24 ± 1.36	
**Optimum ALN**		74.36 ± 1.07	
**Free amygdalin gel**	137.43 ± 1.46	80.17 ± 1.78	1.76 ± 0.04
**Optimum ALN gel**	169.04 ± 1.06	60.45 ± 1.11	3.54 ± 0.03

## Data Availability

Data is contained within the article.
